# A Wake-up Call for Burnout in Portuguese Physicians During the COVID-19 Outbreak: National Survey Study

**DOI:** 10.2196/24312

**Published:** 2021-06-09

**Authors:** Sónia Ferreira, Mafalda Machado Sousa, Pedro Silva Moreira, Nuno Sousa, Maria Picó-Pérez, Pedro Morgado

**Affiliations:** 1 Life and Health Sciences Research Institute (ICVS) School of Medicine University of Minho Braga Portugal; 2 ICVS/3B’s, PT Government Associate Laboratory Braga/Guimarães Portugal; 3 Psychological Neuroscience Lab, CIPsi School of Psychology University of Minho Braga Portugal; 4 2CA-Clinical Academic Center Braga Portugal; 5 P5, Medical Digital Center Braga Portugal

**Keywords:** COVID-19, anxiety, coronavirus, depression, frontline, health care professionals, health care staff, obsessive compulsive disorder, SARS-CoV-2, stress

## Abstract

**Background:**

The COVID-19 outbreak has imposed physical and psychological pressure on health care professionals, including frontline physicians. Hence, evaluating the mental health status of physicians during the current pandemic is important to define future preventive guidelines among health care stakeholders.

**Objective:**

In this study, we intended to study alterations in the mental health status of Portuguese physicians working at the frontline during the COVID-19 pandemic and potential sociodemographic factors influencing their mental health status.

**Methods:**

A nationwide survey was conducted during May 4-25, 2020, to infer differences in mental health status (depression, anxiety, stress, and obsessive compulsive symptoms) between Portuguese physicians working at the frontline during the COVID-19 pandemic and other nonfrontline physicians. A representative sample of 420 participants stratified by age, sex, and the geographic region was analyzed (200 frontline and 220 nonfrontline participants). Moreover, we explored the influence of several sociodemographic factors on mental health variables including age, sex, living conditions, and household composition.

**Results:**

Our results show that being female (β=1.1; *t*=2.5; *P*=.01) and working at the frontline (β=1.4; *t*=2.9; *P*=.004) are potential risk factors for stress. In contrast, having a house with green space was a potentially beneficial factor for stress (β=–1.5; *t*=–2.5; *P*=.01) and anxiety (β=–1.1; *t*=–2.4; *P*=.02).

**Conclusions:**

It is important to apply protective mental health measures for physicians to avoid the long-term effects of stress, such as burnout.

## Introduction

The first case of COVID-19 caused by SARS-CoV-2 was reported in Wuhan, China, in December 2019 [1]. As on December 17, 2020, according to the World Health Organization, after COVID-19 was declared a pandemic, more than 72,000,000 cases were confirmed worldwide, with more than 1,600,000 deaths [2]. In Portugal, the first case was declared on March 2 [3,4], and as on December 17, 2020, a total of 358,296 confirmed cases and 5815 deaths were reported [5]. These numbers correspond to an infection rate of 3.48% and a mortality rate of 0.06% in the total Portuguese population (10,295,909 inhabitants) [6]. At the beginning of the first wave, on March 18, 2020, the Portuguese government declared a state of emergency to avoid virus transmission by applying confinement and social distancing measures [4,7,8]. The number of cases peaked in April 2020 during the first wave and started to decrease until September 2020, when a more severe wave began [4,5].

Recent studies conducted in Portugal during the COVID-19 outbreak reported that mental health status may depend on several protective and risk factors [9,10]. For example, women and younger individuals have greater levels of anxiety, while active workers have a better mental health status.

Medical health workers are among the professionals with a high risk of infection and demanding work conditions, involving lengthy and stressful shifts and situations involving life-or-death decision-making. Thus, they are prone to burnout syndrome and fatigue [11,12]. Recent studies conducted in China [13-19], Iran [20], and the United States [21] during the COVID-19 outbreak reported elevated levels of depression, anxiety, distress, and insomnia among health care workers, namely physicians and nurses. Moreover, certain literature reviews have further supported the impact of COVID-19 on the symptoms of anxiety and depression among health care professionals [22,23]. They have reported a prevalence rate between 18% and 70% for anxiety and between 17% and 40% for depression. Additionally, being a woman, having higher contact with patients with COVID-19, and working in more affected areas were considered risk factors for the psychosocial impact of this disease. Protective factors were associated with support from the family and health systems (eg, training and protective equipment).

Current recommendations and information from the media to avoid touching contaminated surfaces, to engage in frequent cleaning and washing behaviors, and to respect social distancing measures may exacerbate obsessive compulsive (OC) symptoms, namely the fear of contamination and excessive washing [24-28]. Health care professionals are required to work on site and have contact with patients with COVID-19, thus increasing their susceptibility to higher OC symptoms. Few studies have assessed OC symptoms in health professionals during the COVID-19 outbreak [17,18], thus indicating an increase in OC symptomatology.

In this study, we intend to investigate whether Portuguese physicians working at the frontline during the COVID-19 pandemic have worse mental health outcomes (depression, anxiety, stress, and OC symptoms) than their counterparts who are not at the frontline. Additionally, we aimed to explore what sociodemographic factors are potential risk or protective factors for mental health in this sample of health professionals. To our knowledge, this is the first study to explore the psychological impact of the COVID-19 pandemic on Portuguese health care professionals. Our findings may help develop and implement measures to support these professionals during the pandemic.

## Methods

### Study Design

The sample was selected by 2Logical (Lisbon, Portugal). The sample was randomly selected from the company’s main database with information regarding all active physicians in Portugal, to create a final representative sample stratified by age, sex, and geographic region (n=549). A phone-based survey was first conducted with this sample to select participants willing to participate in the study. Consenting participants were invited to take a web-based survey during May 4-25, 2020, during the first wave of the COVID-19 pandemic in Portugal (English version available in Multimedia Appendix 1). At the time, 813 of the 22,749 total infected patients were receiving treatment for COVID-19 at the hospital, with 143 patients receiving intensive care [5]. Since Portugal has a total of 24,000 hospital beds available, including 430 beds in the intensive care unit, the health services were not operating at maximum capacity at the time [4,7]. However, Portugal has a low ratio of nurses per inhabitant, which may contribute to the burden of health care professionals during the pandemic [4,7]. Health care professionals from different fields (eg, internal medicine, anesthesiology, and pneumology) were assigned to intensive care services at this time [29]. Moreover, several emergency service units and COVID-19 community dedicated areas were established to treat patients [7]. Outpatient health care centers continued operating by replacing some presential appointments with telephonic or email contacts and by helping with the diagnosis, treatment, and follow-up of patients with COVID-19 [30,31].

Verbal informed consent was obtained from all subjects. All study procedures comply with the ethical standards of the relevant national and institutional committees on human experimentation and with the tenets of the 2008 revision of the Helsinki Declaration of 1975. All procedures were approved by the Ethical Committee for Life Sciences of the University of Minho (Braga, Portugal; approval# 014/2020). Participants were not compensated for their participation in this study (Portuguese law# 21/2014).

The survey assessed information on age, sex, marital status, and geographic region of residence. The geographic region was categorized into low- and high-risk regions, based on the number of COVID-19 cases (high-risk regions were characterized as having ≥1,000 cases).

Data on living conditions were also acquired: if a participant was displaced from his/her regular habitation, type of current housing (apartment or house), house characteristics including the presence of green spaces and a balcony, the number of house habitants, and the presence of infants, children, teenagers, adults, elders, and pets in the current housing.

Additionally, the survey measured the levels of depression, anxiety, and stress with the 21-items Depression, Anxiety, and Stress Scale (DASS-21) and OC symptoms with the Obsessive-Compulsive Inventory-Revised (OCI-R). The DASS-21 scale assesses symptoms experienced in the prior week. This scale has 21 items with the following response options ranging 0=“did not apply to me at all” to 3=“applied to me very much or most of the time.” The total score varies between 0 and 63. Each subscale (depression, anxiety, and stress) has 7 items and a total score ranging 0-21. Severe depression, anxiety, and stress symptoms correspond to scores higher than 10, 7, and 12, respectively [32]. The OCI-R measures OC symptoms in the previous month. This scale has 18 items divided into 6 categories (3 items each): hoarding, checking, ordering, neutralizing, washing, and obsessing. The answer for each item ranges from 0=“not at all” to 4=“extremely.” The total score varies between 0 and 72, and scores above 20 indicate severe symptomatology [33].

Finally, participants were asked if they were working at the frontline and had direct contact with patients with COVID-19 and if they were currently in a quarantine period. Accordingly, participants were divided into two groups: participants working at the frontline during the COVID-19 pandemic (FRONT) and nonfrontline workers (NFRONT).

### Statistical Analysis

Statistical analyses were conducted using the JASP software (version 0.12.2.0; JASP Team, University of Amsterdam). *P* values under .05 were considered significant.

For scalar variables, we assessed the assumptions of normality (the Shapiro–Wilk test) and homogeneity of variances (the Levene test) in each group. Between-group differences in parametric variables were estimated with the 2-tailed independent samples *t* test. The Mann–Whitney *U* test was applied for nonparametric variables. For categorical variables, we used the chi-square test to assess differences between groups.

We used multiple linear regression models to investigate which variables explained DASS-21 depression, anxiety, and stress scores and the OCI-R total score. We analyzed the following independent variables: age, sex, group, marital status, geographic region, house type, house green space, house balcony, number of house habitants, house infants, house children, house teenagers, house adults, house elders, and house pets. Since most of the sample was not under quarantine and was not displaced from their regular habitation, these variables were not included in the regression models (Table 1). Normality, linearity, and homoscedasticity assumptions were visually assessed with Q-Q plots and residuals vs predicted plots. The Durbin-Watson value was used to assess residuals correlations, and the tolerance and variance inflation factor values were analyzed to check for multicollinearity.

## Results

A total of 549 physicians were contacted to participate in the study, of whom 420 (76.5%) responded to the survey. The participants who refused to be included in the study mainly declared a lack of interest or time. The physicians included in the study belonged to different specialties: general and family practice (n=155, 36.9%), internal medicine (n=62, 14.8%), pneumology (n=32, 7.6%), pediatrics (n=23, 5.5%), oncology (n=20, 4.8%), cardiology (n=18, 4.3%), psychiatry (n=17, 4.0%), gynecology (n=13, 3.1%), intensive medicine (n=12, 2.9%), infectiology (n=11, 2.6%), and other specialties (n=57, 13.6%) including hematology, endocrinology, immunoallergology, gastroenterology, dermatology, urology, neurology, rheumatology, orthopedics, and ophthalmology. In total, 68% of the participants worked in public settings, 11% in the private sector, and 21% in both public and private settings. A total of 200 participants were included in the FRONT group and 220 in the NFRONT group. Table 1 lists the main variables for both groups and the total sample.

When analyzing differences between the FRONT and NFRONT groups, we obtained significant outcomes for age, sex, house type, marital status, and the presence of children and adults in the house (Table 1). The FRONT group was younger than the NFRONT group (1 participant from the FRONT group was excluded from this analysis owing to incorrect data entered for age). Moreover, the FRONT group had a higher number of females than males in contrast with the NFRONT group. More frontline workers lived in an apartment rather than a house in contrast with the NFRONT group. Additionally, the number of frontline participants in a marriage or partnership was lower and the number of divorced and single individuals was higher than those in the NFRONT group. The number of participants living with children and adults was lower in the FRONT than in the NFRONT groups. The observed sociodemographic differences between the FRONT and NFRONT groups might be associated with age differences; older individuals might already be married or live with a partner and have children and a bigger house.

**Table 1:** Demographic data of the Portuguese physicians at the frontline and those not at the frontline during the COVID-19 pandemic (May 4-25, 2020), along with the statistics for between-group comparisons (Mann–Whitney *U* values for scalar variables and *χ*^2^ values for categorical variables).

Characteristic		Frontline workers (n=200)	Nonfrontline workers (n=220)	Total (n=420)	Between-group statistics	
					Statistics	*P* values
Age (years), median (IQR)		47.0 (22.0)^a^	60.0 (21.2)	53.0 (23.0)	*U*=29,568.5; RBC^b^=0.3^c^	<.001
**Sex, n (%)**					*χ*^2^(*1*)=4.1	.04^c^
	Female	107 (53.5)	96 (43.6)	203 (48.3)	N/A^d^	N/A
	Male	93 (46.5)	124 (56.4)	217 (51.7)	N/A	N/A
**In quarantine, n (%)**					*χ*^2^(*1*)=0.5	.48
	Yes	2 (1.0)	4 (1.8)	6 (1.4)	N/A	N/A
	No	198 (99.0)	216 (98.2)	414 (98.6)	N/A	N/A
**Geographic region, n (%)**					*χ*^2^(*1*)=2.1	.14
	High-risk	107 (53.5)	102 (46.4)	209 (49.8)	N/A	N/A
	Low-risk	93 (46.5)	53.6 (118)	211 (50.2)	N/A	N/A
**House displacement, n (%)**					*χ*^2^(*1*)=0.4	.52
	Yes	16 (8.0)	14 (6.4)	30 (7.1)	N/A	N/A
	No	184 (92.0)	206 (93.6)	390 (92.9)	N/A	N/A
**House type, n (%)**					*χ*^2^(*1*)=5.8	.02
	House	82 (41.0)	116 (52.7)	198 (47.1)	N/A	N/A
	Apartment	118 (59.0)	104 (47.3)	222 (52.9)	N/A	N/A
**Green space around the house, n (%)**					*χ*^2^(*1*)=3.6	.06
	Yes	110 (55.0)	141 (64.1)	251 (59.8)	N/A	N/A
	No	90 (45.0)	79 (35.9)	169 (40.2)	N/A	N/A
**Balcony in the house, n (%)**					*χ*^2^(*1*)=0.4	.51
	Yes	172 (86.0)	194 (88.2)	366 (87.1)	N/A	N/A
	No	28 (14.0)	26 (11.8)	54 (12.9)	N/A	N/A
**Marital status, n (%)**					*χ*^2^(*3*)=8.4	.04^c^
	Married/partnership	136 (68.0)	167 (75.9)	303 (72.1)	N/A	N/A
	Divorced	38 (19.0)	27 (12.3)	65 (15.5)	N/A	N/A
	Single	26 (13.0)	22 (10.0)	48 (11.4)	N/A	N/A
	Widow	0 (0.0)	4 (1.8)	4 (0.9)	N/A	N/A
Number of house habitants, median (IQR)		2.0 (2.0)	2.0 (2.0)	2.0 (2.0)	*U*=23,987.5; RBC=0.09	.10
**House infants, n (%)**					*χ*^2^(*1*)=3.5	.06
	Yes	13 (6.5)	26 (11.8)	39 (9.3)	N/A	N/A
	No	187 (93.5)	194 (88.2)	381 (90.7)	N/A	N/A
**House children, n (%)**					*χ*^2^(*1*)=5.9	.02^c^
	Yes	34 (17.0)	59 (26.8)	93 (22.1)	N/A	N/A
	No	166 (83.0)	161 (73.2)	327 (77.9)	N/A	N/A
**House teenagers, n (%)**					*χ*^2^(*1*)=0.014	.91
	Yes	29 (14.5)	31 (14.1)	60 (14.3)	N/A	N/A
	No	171 (85.5)	189 (85.9)	360 (85.7)	N/A	N/A
**House adults, n (%)**					*χ*^2^(*1*)=5.0	.03^c^
	Yes	134 (67.0)	169 (76.8)	303 (72.1)	N/A	N/A
	No	66 (33.0)	51 (23.2)	117 (27.9)	N/A	N/A
**House elders, n (%)**					*χ*^2^(*1*)=0.2	.64
	Yes	39 (19.5)	39 (17.7)	78 (18.6)	N/A	N/A
	No	161 (80.5)	181 (82.3)	342 (81.4)	N/A	N/A
**House pets, n (%)**					*χ*^2^(*1*)=0.01	.92
	Yes	101 (50.5)	110 (50.0)	211 (50.2)	N/A	N/A
	No	99 (49.5)	110 (50.0)	209 (49.8)	N/A	N/A

^a^One participant with missing information (incorrect data entry).

^b^RBC: rank biserial correlation effect size.

^c^Values are significant.

^d^N/A: not applicable.

Variables for age and sex differences between groups were used as covariates when assessing differences between FRONT and NFRONT groups for depression, anxiety, stress, and OC scores. The results of the analysis of covariance are displayed in Table 2. One participant from the FRONT group was excluded from this analysis owing to an incorrect data entry for age. We observed a significant effect of sex on the DASS-21 anxiety score. Female participants presented higher anxiety levels than males. Additionally, we observed significant effects of group, age, and sex on the DASS-21 stress score. FRONT physicians displayed higher stress levels than NFRONT participants. Younger participants had higher stress levels. Finally, females had higher stress levels than males.

**Figure 1:**
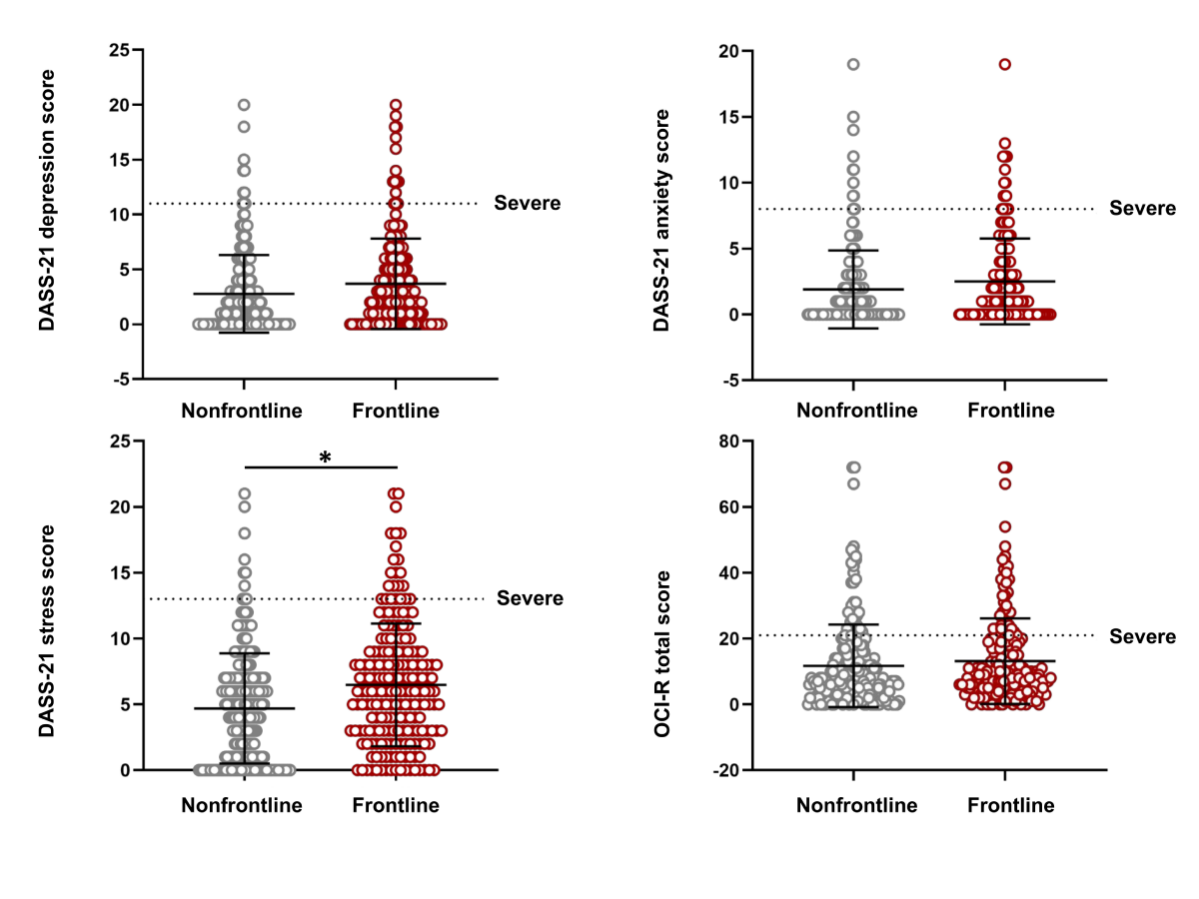
DASS-21 and OCI-R scores of frontline (n=200) and nonfrontline (n=220) Portuguese physicians during the COVID-19 pandemic (May 4-25, 2020). Lines represent mean (SD) values. Points above the dotted line represent participants with severe symptoms. *Statistically significant differences between nonfrontline and frontline groups. DASS-21: 21-items Depression, Anxiety, and Stress Scale; OCI-R: Obsessive-Compulsive Inventory-Revised.

Regarding the DASS-21 depression score, 15 (7.5%) participants in the FRONT group and 10 (4.5%) in the NFRONT group had severe symptoms (total sample=25, 5.9%). Regarding the DASS-21 anxiety score, 18 (9.0%) FRONT physicians presented severe symptoms compared to 13 (5.9%) NFRONT physicians (total sample=31, 7.4%). Regarding the DASS-21 stress score, 23 (11.5%) participants in the FRONT group had severe symptoms compared to 10 (4.5%) in the NFRONT group (total sample=33, 7.9%). Lastly, 39 (19.5%) FRONT physicians had severe OCI-R total scores as opposed to 36 (16.4%) in the NFRONT group (total sample=75, 17.9%) (Figure 1).

**Figure 2:**
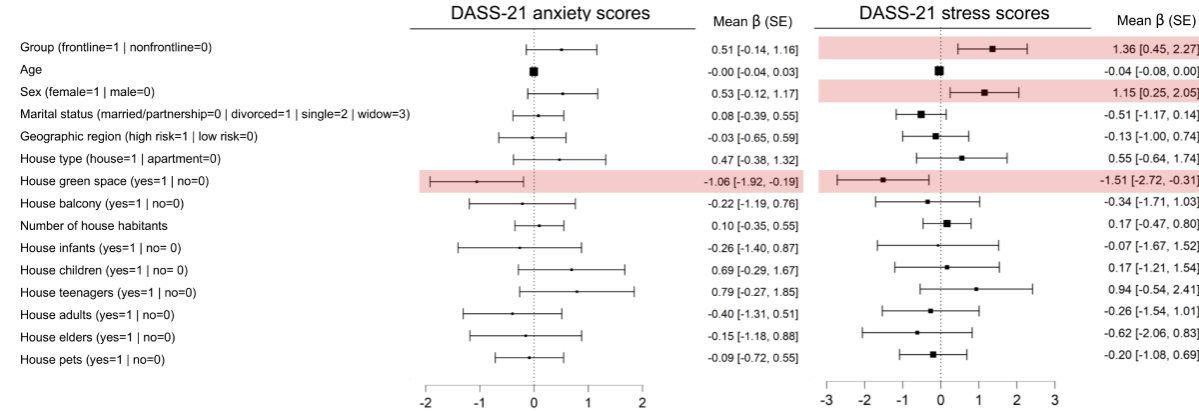
Significant variables in regression models for DASS-21 anxiety and DASS-21 stress scores of Portuguese physicians (n=420) during the COVID-19 pandemic (May 4-25, 2020). DASS-21 depression and OCI-R total models did not reach significance. The forest plot represents the mean unstandardized β (SE) values. The red boxes indicate statistical significance. One participant was excluded because of missing information for age (incorrect data entry). DASS-21: 21-items Depression, Anxiety, and Stress Scale; OCI-R: Obsessive-Compulsive Inventory-Revised.

Regression models based on the DASS-21 anxiety (*F*_15,403_=1.84; *P*=.03; *R*^2^=0.06) and stress (*F*_15,403_=3.64; *P*<.001; *R*^2^=0.12) scores were significant, while those based on the DASS-21 depression (*F*_15,403_=1.54; *P*=.09; *R*^2^=0.05) and OCI-R (*F*_15,403_=0.64; *P*=.85; *R*^2^=0.02) scores did not reach significance. Regarding regression models that reached significance, having a green space at home helped reduce the DASS-21 anxiety (β=–1.06, SE 0.44; standardized β=–0.17; *t*=-2.40; *P*=.02) and stress (β=–1.51, SE 0.61; standardized β=–0.16; *t*=–2.46; *P*=.01) scores. Moreover, being female (β=1.15, SE 0.46; standardized β=0.13; *t*=2.50; *P*=.01) and being a frontline physician (β=1.36, SE 0.46; standardized β=0.15; *t*=2.93; *P*=.004) was associated with a high DASS-21 stress score (Figure 2).

Considering the effects of sex on the outcomes of the analysis of covariance and regression models for anxiety and stress, we used the regression models for DASS-21 anxiety and stress separately for males and females. DASS-21 anxiety models for males (*F*_14,202_=0.96; *P*=.50; *R*^2^=0.06) and females (*F*_14,187_=1.46; *P*=.13; *R*^2^=0.10) did not reach significance. The DASS-21 stress model reached significance for females (*F*_14,187_=2.45; *P*=.008; *R*^2^=0.14) but not for males (*F*_14,202_=1.41; *P*=.15; *R*^2^=0.09). Female FRONT physicians had higher levels of stress than their NFRONT counterparts (β=1.59, SE 0.65; standardized β=0.18; *t*=2.45; *P*=.02).

**Table 2:** Results of the analysis of covariance between Portuguese physicians at the frontline (n=200) and those not at the frontline (n=220) during the COVID-19 pandemic (May 4-25, 2020) with respect to the DASS-21^a^ and OCI-R^b^ scales, using age and sex as covariates.

Parameter			Values, mean (SD)	Between-group statistics	
				Statistics	*P* values
**DASS-21 depression score**					
	**Group**			*F* (*1,415*)=2.7; *η*^2^=0.065	.10
		Frontline^c^	3.69 (4.12)	N/A^d^	N/A
		Nonfrontline	2.78 (3.54)	N/A	N/A
	Age		N/A	*F* (*1,415*)=1.9; *η*^2^=0.004	.17
	**Sex**			*F* (*1,415*)=0.7; *η*^2^=0.001	.41
		Female	3.54 (3.75)	N/A	N/A
		Male	2.90 (3.92)	N/A	N/A
**DASS-21 anxiety score**					
	**Group**			*F* (*1,415*)=1.8; *η*^2^=0.004	.18
		Frontline	2.50 (3.25)	N/A	N/A
		Nonfrontline	1.89 (2.96)	N/A	N/A
	Age		N/A	*F* (*1,415*)=1.4; *η*^2^=0.003	.24
	**Sex**			*F* (*1,415*)=4.7; *η*^2^=0.011^e^	.03
		Female	2.63 (3.20)	N/A	N/A
		Male	1.77 (2.98)	N/A	N/A
**DASS-21 stress score**					
	**Group**			*F* (*1,415*)=8.3; *η*^2^=0.019^e^	.004
		Frontline	6.47 (4.65)	N/A	N/A
		Nonfrontline	4.69 (4.19)	N/A	N/A
	Age		N/A	*F* (*1,415*)=6.8; *η*^2^=0.016^e^	.009
	**Sex**			*F* (*1,415*)=7.5; *η*^2^=0.017^e^	.007
		Female	6.45 (4.40)	N/A	N/A
		Male	4.69 (4.44)	N/A	N/A
**OCI-R total score**					
	**Group**			*F* (*1,415*)=1.8; *η*^2^=0.004	.18
		Frontline	13.12 (13.00)	N/A	N/A
		Nonfrontline	11.70 (12.52)	N/A	N/A
	Age		N/A	*F* (*1,415*)=0.9; *η*^2^=0.002	.35
	**Sex**			*F* (*1,415*)=0.4; *η*^2^=0.001	.51
		Female	12.68 (12.33)	N/A	N/A
		Male	12.10 (13.17)	N/A	N/A

^a^DASS-21: 21-items Depression, Anxiety, and Stress Scale.

^b^OCI-R: Obsessive-Compulsive Inventory-Revised.

^c^One participant with missing information (incorrect data entry).

^d^N/A: not applicable.

^e^Values are significant.

## Discussion

### Principal Findings

In this study, we investigated whether the mental health of physicians working at the frontline during the COVID-19 pandemic is affected in comparison to other physicians. We found that frontline physicians have increased levels of stress. Furthermore, female physicians presented higher levels of stress and anxiety. Younger physicians also displayed augmented stress levels. Concurrent with these results, being female and working at the frontline are potential risk factors for stress, and having a house with green space is a potential protective factor for stress and anxiety.

These findings are consistent with those of our previous study on the general Portuguese population, which reported that being male, being older, and living in a house with green space appeared to be beneficial for mental health during the COVID-19 pandemic [10]. Moreover, previous studies have reported that women and younger individuals have a higher risk of mental disorders, including anxiety and mood disorders [34,35]. Furthermore, previous studies have reported negative associations between mental health variables (eg, stress and anxiety) and age among health care workers, indicating that older individuals might be more resilient to the psychological impact of COVID-19 [20,36,37]. Moreover, older physicians might cope better with stressful situations because they have more years of experience. On the other hand, older physicians might have been assigned to lower-exposure wards, considering the higher risk of more severe manifestations of COVID-19 with an older age [29]. Indeed, NFRONT participants were older than their FRONT counterparts in our study. Of note, age was not a significant variable in the stress regression model; this finding should be interpreted with caution. Parallel with our results, several recent studies reported that female medical workers have higher levels of anxiety and stress [13,18,20,21,36-38]. In addition to working outside of the house, women usually have more responsibilities within their household and with childcare than men. Moreover, they rely more on social support as a coping strategy for stress [39-41]. Thus, female physicians may have been exposed to more stressors during the COVID-19 pandemic because they might need to reconcile home, childcare, and work duties while having less social support from family and friends to follow social isolation guidelines, along with less support from spouses and their workplaces. Additionally, as a protective measure, schools and daycare centers for children were closed during the first wave of COVID-19 in Portugal [42], thus aggravating the lack of childcare support for women and possibly affecting their work schedule. On the other hand, in Portugal, more female physicians work in specialties including general and family practice and anesthesiology, internal medicine, and pneumology [43], which involve high contact with patients, while male physicians are more predominant in surgical fields, where several services were canceled during the pandemic [29]. This might also have contributed to the observed levels of anxiety and stress among female physicians. Lastly, having a house with green spaces, such as a garden, might help increase physical activity and provide outdoor relaxation periods, thus contributing to better mental health [44,45]. Thus, the effect observed for this variable might be a surrogate for high-quality leisure time.

Furthermore, in comparison with our previous results [10], frontline physicians presented a higher prevalence of severe OC symptoms (19.5%) than the general population (12.4%). However, we did not observe significant differences between FRONT and NFRONT physicians with respect to OC symptoms. Interestingly, the prevalence of severe OC symptoms in NFRONT physicians (16.4%) was also higher than that in the general population. Thus, COVID-19 might have an impact on OC symptoms among physicians in general, concurrent with previous reports [17,18]. The fear of self-infection or disease transmission among family members or coworkers because of working at health facilities with a high risk of infection may boost excessive washing and cleaning behaviors or contamination-related obsessive thoughts.

Furthermore, frontline workers in our study showed higher stress levels than other physicians and a higher prevalence of severe stress symptoms (11.5%) than the general population (9.3%) [10]. Previous studies have also reported increases in stress [36,37,46], distress [13], and burnout [11,12] symptoms among medical workers during the COVID-19 pandemic. These and other studies suggest that the fear of self-infection and disease transmission to family members, concern or grief for affected coworkers, social distance from family members, lack of proper COVID-19–related training and protective equipment, longer working hours, lack of sleep, and difficult life-or-death decision-making might be factors contributing to increased stress [38,46-51]. Other studies have also observed positive correlations between stress and the fear of infection and between stress and burnout symptoms among health care professionals [52]. Furthermore, previous outbreaks of other infectious diseases (eg, severe acute respiratory syndrome, Middle East respiratory syndrome, and A/H1N1 influenza) were associated with an increase in stress, distress, and burnout symptoms among health care professionals [53,54]. Long-term effects of augmented stress levels may translate to posttraumatic stress disorder and burnout, especially among frontline physicians [47,48,50,51,53,55].

Studies conducted in several countries have reported that health care professionals have elevated levels of anxiety and depression during the COVID-19 pandemic [22,23]. We did not observe evidence of increases in the symptoms of severe anxiety and depression in our sample when compared to a previous study on the general Portuguese population [10], and frontline workers were not different from other physicians in terms of these symptoms. These findings may be explained by the lower number of COVID-19 cases and the subsequent occurrence of the disease in Portugal when compared to countries that were among the first to be affected by this disease.

### Limitations

This study has some limitations. We used a cross-sectional design, which prevents the inference of causality. The selection of participants was not free of putative bias because the physicians who consented to participate in the study might have better mental health indicators or a reduced workload (eg, by not working at the frontline) than the ones who refused to participate. Additionally, our conclusions cannot be applied to other health care professionals such as nurses who have had direct contact with patients with COVID-19. Since few physicians in our sample were in a quarantine period, we could not study the impact of this variable on mental health. Finally, other factors that were not considered in our analysis might have had a significant impact on mental health, including workload [19,21,46,52], history of psychiatric disorders [56], and the use of coping strategies (eg, use of substances, exercise, and mindfulness) [14,46]. Furthermore, we did not determine how long these physicians have been working at the frontline during the COVID-19 pandemic, whether they switched to other wards during the pandemic, whether they received psychological or psychiatric support [57], or whether they were facing difficulties in daily functioning at work or during social activities [58,59]. Thus, our findings should be interpreted with caution in consideration of these limitations.

### Conclusions

This is the first study to provide evidence of increased stress levels in Portuguese physicians at the frontline of during the COVID-19 pandemic. We observed that female physicians are more susceptible to stress. Prolonged exposure to COVID-19–related stressors may lead to burnout syndrome. Thus, our results potentially provide essential guidelines for future preventive actions by health care systems, namely the establishment of proper rest periods, control of excessive workloads, the supply of basic needs (eg, personal safety and childcare), development of pandemic-related training programs, and the incorporation of protective measures for mental health (eg, virtual mindfulness-based interventions). Additionally, social contact with family and friends should not be overpowered by occupational demands [47-50].

## Appendix

Multimedia Appendix 1 Table S1 - new supplementary table with survey information (round 2).

## Abbreviations

DASS-21: 21-items Depression, Anxiety, and Stress Scale
FRONT: frontline physicians during the COVID-19 pandemic
OC: obsessive compulsive
OCI-R: Obsessive-Compulsive Inventory-Revised
NFRONT: nonfrontline physicians
