# Supplementary material for: A Wake-up Call for Burnout in Portuguese Physicians During the COVID-19 Outbreak: National Survey Study
Source: JMIR Public Health Surveill. 2021 Jun 9;7(6):e24312. doi: 10.2196/24312 (PMC8191732; doi:10.2196/24312)
Supplement: Multimedia Appendix 1 [file publichealth_v7i6e24312_app1.docx]

| **Table S1 English version of the online survey applied to Portuguese physicians working at the frontline (*n* = 200) and not at the frontline (*n* = 220) of COVID-19 pandemic (4 to 25 of May 2020).** | | |
| --- | --- | --- |
| Number | Question | Answering options |
| 1 | Birth year | - |
| 2 | Sex | Female  Male |
| 3 | Work district | Aveiro, Beja, Braga, Bragança, Castelo Branco, Coimbra, Évora, Faro, Guarda, Leiria, Lisboa, Portalegre, Porto, Santarém, Setúbal, Viana do Castelo, Vila Real, Viseu, Açores, and Madeira |
| 4 | Marital status | Married/partnership  Divorced  Single  Widow |
| 5 | Are you at the frontline of COVID-19 with contact with COVID-19 patients? | Yes  No |
| 6 | Are you under a mandatory quarantine period after suspicion of COVID-19 infection? | Yes  No |
| 7 | Are you displaced from your regular home? | Yes  No |
| 8 | Type of house where you currently live in | Detached house  Semi-detached house  Apartment |
| 9 | Does it have an outdoor green space? | Yes  No |
| 10 | Does it have a balcony, a deck, or a courtyard? | Yes  No |
| 11 | Number of people with whom you share your house | - |
| 12 | What is their age range? | Babies (0-3 years old)  Children (4-12 years old)  Teenagers (13-17 years old)  Adults (18-64 years old)  Elders (≥ 65 years old) |
| 13 | Do you have pets at home? | Yes  No |
